# A Deep Learning Based Framework for Diagnosing Multiple Skin Diseases in a Clinical Environment

**DOI:** 10.3389/fmed.2021.626369

**Published:** 2021-04-16

**Authors:** Chen-Yu Zhu, Yu-Kun Wang, Hai-Peng Chen, Kun-Lun Gao, Chang Shu, Jun-Cheng Wang, Li-Feng Yan, Yi-Guang Yang, Feng-Ying Xie, Jie Liu

**Affiliations:** ^1^Department of Dermatology, State Key Laboratory of Complex Severe and Rare Diseases, Peking Union Medical College Hospital, Chinese Academy of Medical Sciences and Peking Union Medical College, Beijing, China; ^2^DeepWise AI Lab, Beijing, China; ^3^Image Processing Center, School of Astronautics, Beihang University, Beijing, China

**Keywords:** artificial intelligence, deep learning, convolutional neural networks, dermatology, skin diseases, skin imaging, dermoscopy

## Abstract

**Background:** Numerous studies have attempted to apply artificial intelligence (AI) in the dermatological field, mainly on the classification and segmentation of various dermatoses. However, researches under real clinical settings are scarce.

**Objectives:** This study was aimed to construct a novel framework based on deep learning trained by a dataset that represented the real clinical environment in a tertiary class hospital in China, for better adaptation of the AI application in clinical practice among Asian patients.

**Methods:** Our dataset was composed of 13,603 dermatologist-labeled dermoscopic images, containing 14 categories of diseases, namely lichen planus (LP), rosacea (Rosa), viral warts (VW), acne vulgaris (AV), keloid and hypertrophic scar (KAHS), eczema and dermatitis (EAD), dermatofibroma (DF), seborrheic dermatitis (SD), seborrheic keratosis (SK), melanocytic nevus (MN), hemangioma (Hem), psoriasis (Pso), port wine stain (PWS), and basal cell carcinoma (BCC). In this study, we applied Google's EfficientNet-b4 with pre-trained weights on ImageNet as the backbone of our CNN architecture. The final fully-connected classification layer was replaced with 14 output neurons. We added seven auxiliary classifiers to each of the intermediate layer groups. The modified model was retrained with our dataset and implemented using Pytorch. We constructed saliency maps to visualize our network's attention area of input images for its prediction. To explore the visual characteristics of different clinical classes, we also examined the internal image features learned by the proposed framework using t-SNE (t-distributed Stochastic Neighbor Embedding).

**Results:** Test results showed that the proposed framework achieved a high level of classification performance with an overall accuracy of 0.948, a sensitivity of 0.934 and a specificity of 0.950. We also compared the performance of our algorithm with three most widely used CNN models which showed our model outperformed existing models with the highest area under curve (AUC) of 0.985. We further compared this model with 280 board-certificated dermatologists, and results showed a comparable performance level in an 8-class diagnostic task.

**Conclusions:** The proposed framework retrained by the dataset that represented the real clinical environment in our department could accurately classify most common dermatoses that we encountered during outpatient practice including infectious and inflammatory dermatoses, benign and malignant cutaneous tumors.

## Introduction

Dermatology is a branch of clinical medicine of which the diagnosis and treatment monitor greatly rely on the morphology of various cutaneous lesions. The traditional diagnostic process of dermatoses is thus based on the integration of patients' medical history, clinical manifestation, dermoscopic images and sometimes histopathological evaluation by the dermatologists. However, the training of becoming an experienced dermatologist is time-consuming. Furthermore, cutaneous diseases are vast in type and can be very similar in appearance under human eyes, leading to the difficulties in accurate and effective diagnosis. Recent advances on artificial intelligence (AI), particularly convolutional neural networks (CNN) based deep learning algorithms, have made it possible to learn the most predictive features of diseases directly from medical images given a large dataset of labeled examples (1, 2). Esteva et al. proposed a dermatologist level classification of skin cancer via fine-tuning a pre-trained Inception-v3 network (3). Menegola et al. also conducted experiments comparing training from scratch with fine-tuning of pre-trained networks on images with skin lesions (4). Their work showed that fine-tuning of pre-trained networks worked better than training from scratch. Numerous researches based on AI using dermoscopic and non-dermoscopic images have attempted to apply this technology in the dermatological field, including segmentation and classification of melanocytic tumors, keratinocyte tumors, ulcers, psoriasis and other inflammatory dermatoses (5–11). Some of the AI models showed astonishing diagnostic capacity that could reach or even surpass a dermatologist level (3, 7, 12, 13). Therefore, currently those computer-aided diagnostic model applications are tied with great hope and promise in screening and helping to diagnose cutaneous diseases.

Although these studies have achieved acceptable accuracy in specific diseases with different models trained by various databases, researches under real clinical settings are scarce. Additionally, previous studies applying AI models in real clinical settings showed relatively poorer performance outcomes comparing with that using their own experimental datasets (14, 15). In clinical practice, the most frequently encountered diseases are not limited to the kinds mentioned above, especially in Asian countries like China where the incidence of melanoma is relatively lower. Therefore, for better adaption of the AI application in dermatological field, we investigated the imaging database of the Department of Dermatology, Peking Union Medical College Hospital, and extracted the imaging data of the 14 most frequently encountered dermatoses to form a dataset that represented the real clinical environment of our clinics. The disease categories included melanocytic nevus (MN), seborrheic keratosis (SK), dermatofibroma (DF), keloid and hypertrophic scar (KAHS), basal cell carcinoma (BCC), hemangioma (Hem), port wine stain (PWS), eczema/dermatitis (EAD), psoriasis (Pso), seborrheic dermatitis (SD), rosacea (Rosa), acne vulgaris (AV), lichen planus (LP), and viral warts (VW). We used this dataset to construct a novel framework based on deep learning, aiming to verifying the classification performance of this proposed framework under a more practical and representative circumstance, and further compare its diagnostic accuracy with certificated dermatologists in China.

This novel CNN model we proposed here was on the basis of the EfficientNet-b4 CNN algorithm. We utilized multiple auxiliary prediction modules among different intermediate layers to accumulate discriminative information from different levels of features. The proposed CNN model was trained on 13,603 clinical images from 2,538 patient cases, and had been evaluated on expert-confirmed clinical images grouped into 14 different dermatologist-labeled diagnoses mentioned above. According to the experiment, it outperformed common convolutional networks and was competitive with dermatologists in real-world diagnosis.

## Materials and Methods

### Ethical Approval

We conducted this research according to the ethical tenets of the *Declaration of Helsinki*. And this study was approved by the Medical Ethics Committee of Peking Union Medical College Hospital (NO. JS-2003). Informed written consents were obtained from all the included adult patients or the guardians of juvenile patients.

### Datasets

Our dataset was collected and formed from the imaging database of the Department of Dermatology, Peking Union Medical College Hospital in China from October 2016 to April 2020. All the included patients were Asian with Fitzpatrick skin type III or IV, and their dermoscopic images were consecutively acquired using a digital dermoscopy system (MoleMax HD 1.0 dermoscope, Digital Image Systems, Vienna, Austria) by the same technician to ensure the quality and standardization of the images. Generally, multiple dermoscopic images were captured of a single lesion in different angles or in subsequent follow-up presentations. The annotation process was performed by 2 dermatologists with more than 5-years experience according to the patients' medical history, clinical manifestations and dermoscopic features independently. All the patients with BCC and few ambiguous cases with benign conditions were confirmed by histopathological investigations. Whenever there was a disagreement between the annotators, consensus was reached through discussion or consultant with a third dermatological expert. Cases with unclear or incomplete medical history, whose images were poorly focused, and lesions located on the nail or mucosa areas were excluded. The final dataset for the development of the model and the demographic characteristics of the included patients were shown in Table 1.

**Table 1:** Dataset overview.

**Characteristics**	**Development set**		**Test set of the reader study**	
	**Images**	**Patients**	**Images**	**Patients**
Numbers included in study (*n*)	13,603	2,538	200	200
Mean age (years), mean ± SD (range)	-	57.38 ± 16.81	-	43.55 ± 19.68
Female, *n* (%)	-	1,532 (60.36)	-	123 (61.50)
Lichen planus, *n* (%)	804 (5.91)	126 (4.96)	-	-
Rosacea, *n* (%)	597 (4.39)	80 (3.15)	25 (12.5)	25 (12.5)
Viral warts, *n* (%)	1,110(8.16)	298 (11.74)	25 (12.5)	25 (12.5)
Acne vulgaris, *n* (%)	2,023(14.87)	277 (10.91)	-	-
Keloid and hypertrophic scar, *n* (%)	438 (3.22)	96 (3.78)	-	-
Eczema/dermatitis, *n* (%)	2,440 (17.94)	419 (16.51)	25 (12.5)	25 (12.5)
Dermatofibroma, *n* (%)	343 (2.52)	116 (4.57)	-	-
Seborrheic dermatitis, *n* (%)	767 (5.64)	124 (4.89)	25 (12.5)	25 (12.5)
Seborrheic keratosis, *n* (%)	553 (4.07)	143 (5.63)	25 (12.5)	25 (12.5)
Melanocytic nevus, *n* (%)	1,214 (8.92)	345 (13.59)	25 (12.5)	25 (12.5)
Hemangioma, *n* (%)	200 (14.70)	61 (2.40)	-	-
Psoriasis, *n* (%)	1,707 (12.55)	234 (9.22)	25 (12.5)	25 (12.5)
Port wine stain, *n* (%)	920 (6.76)	112 (4.41)	-	-
Basal cell carcinoma, *n* (%)	487 (3.58)	107 (4.22)	25 (12.5)	25 (12.5)

### CNN and Deep Learning Algorithm

**Figure 1:**
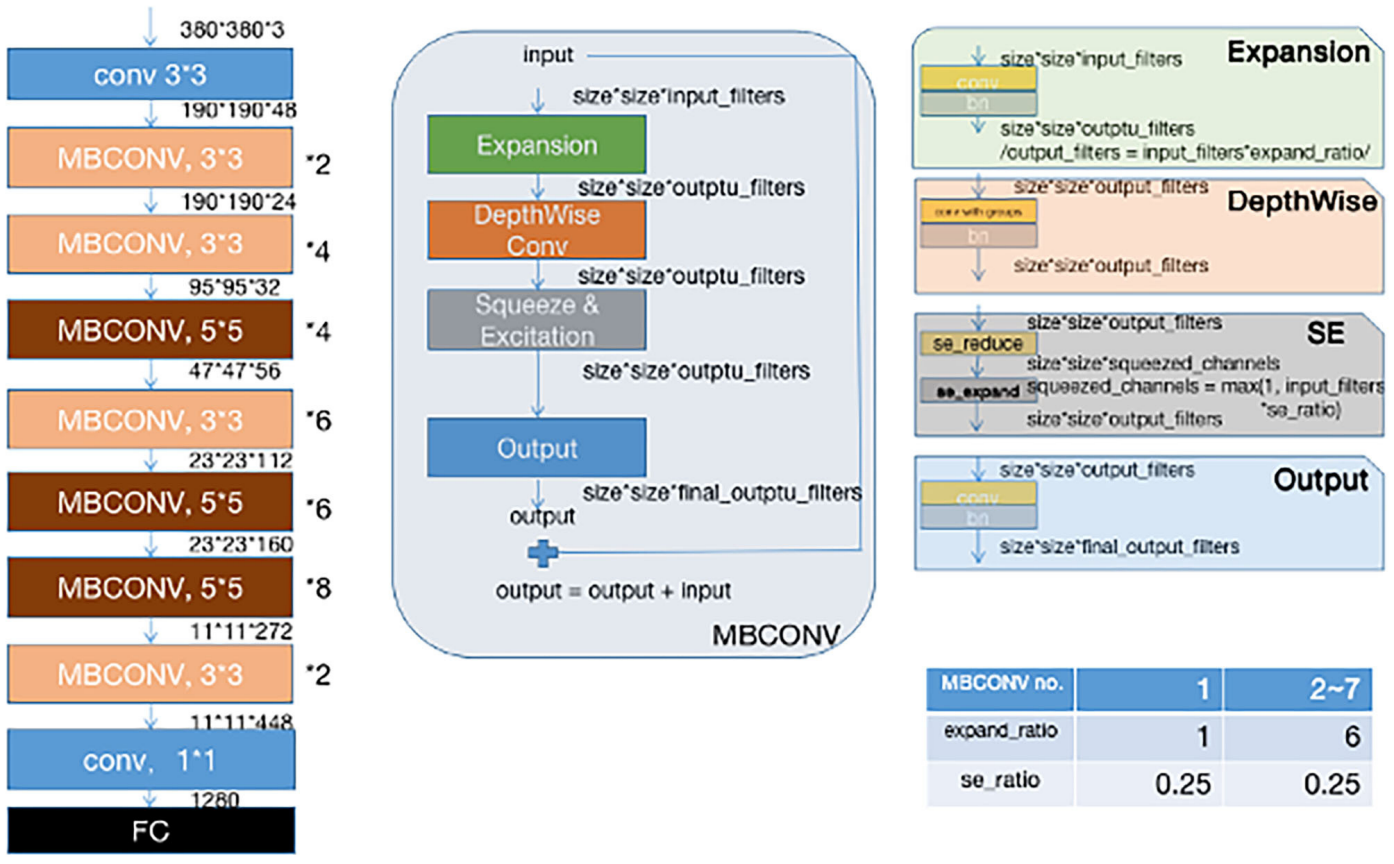
Original efficientnet-b4 architecture.

**Figure 2:**
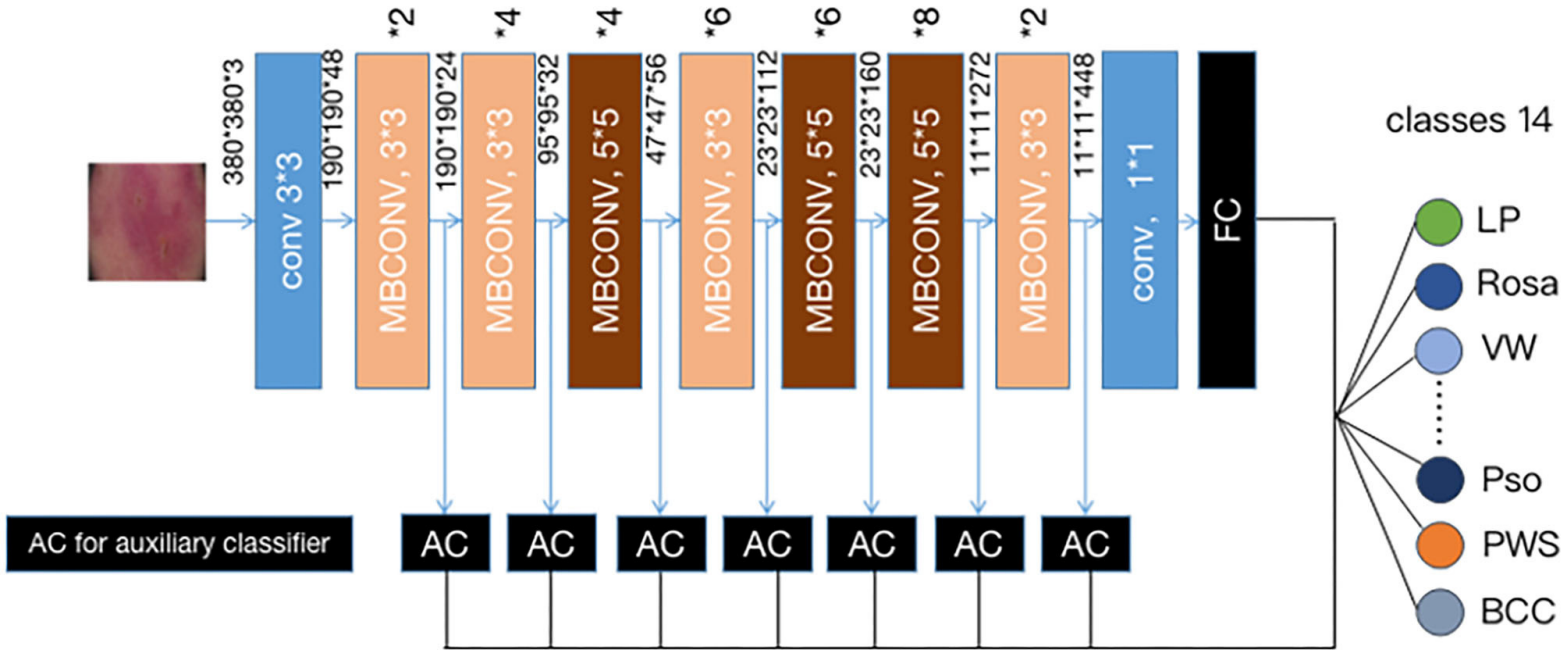
Our modified (19) EFFICIENTNET-b4 architecture. Data flow is from left to right: a dermoscopic image is put into the network and finally transformed into a probability distribution over clinical classes of skin disease using our modified EfficientNet-b4 architecture pretrained on the ImageNet dataset and fine-tuned on our own dataset of 13,603 dermoscopic images in 14 categories.

Instead of training a new model from scratch, we applied a fine-tuning strategy directly on pre-trained models via a multi-step retraining strategy with our dataset. In this strategy, we gradually unfroze the layer weights in steps with the first few layers being unfrozen last. In these steps, we reduced learning rates progressively from 1e-3 to 1e-5 and kept other parameters unchanged. As shown in Figure 1, we applied Google's EfficientNet-b4 (16) with pre-trained weights on the 2015 ImageNet dataset (17) with 1,000 object categories as the backbone of the proposed deep learning framework. The final fully-connected classification layer was replaced with 14 output neurons of our classifier. We also add seven auxiliary classifiers at the end of each intermediate layer (18) to make the model learn classification information from different level of features as shown in Figure 2.

Our deep learning network was implemented using Pytorch. Initially, a global learning rate 0.001 was used and it decayed to 0.0001 at epoch 25. We used a mini-batch gradient descent with a momentum 0.9 as the model parameters optimizer. In training, each image was resized to 380^*^380 pixels in RGB channels, the optimized input size of EfficientNet-b4. For each epoch, each image would be rotated from −30° to 30° randomly, together with 50% probabilities for vertical and horizontal flipping. Color constancy method was also applied to eliminate the color deviation.

The raw output of our classifiers (seven auxiliary classifiers plus 1 final classifier) was summarized by 14 classification neurons. And then an argmax operation was used to find the most likely classification of the input image. The formula is as follows:

$$\begin{array}{l} \text{classification}=\text{argmax}\left(\overset{\text{Classifiers\_number}}{\underset{\text{i}=1}{\sum }}\text{outpu}{\text{t}}_{\text{i}}\right)\; \end{array}$$

where *output*_*i*_ is the classification vector with dimension (1, 14) and the sum of eight classification vectors is an element-wise summation.

### Saliency Maps

**Figure 3:**
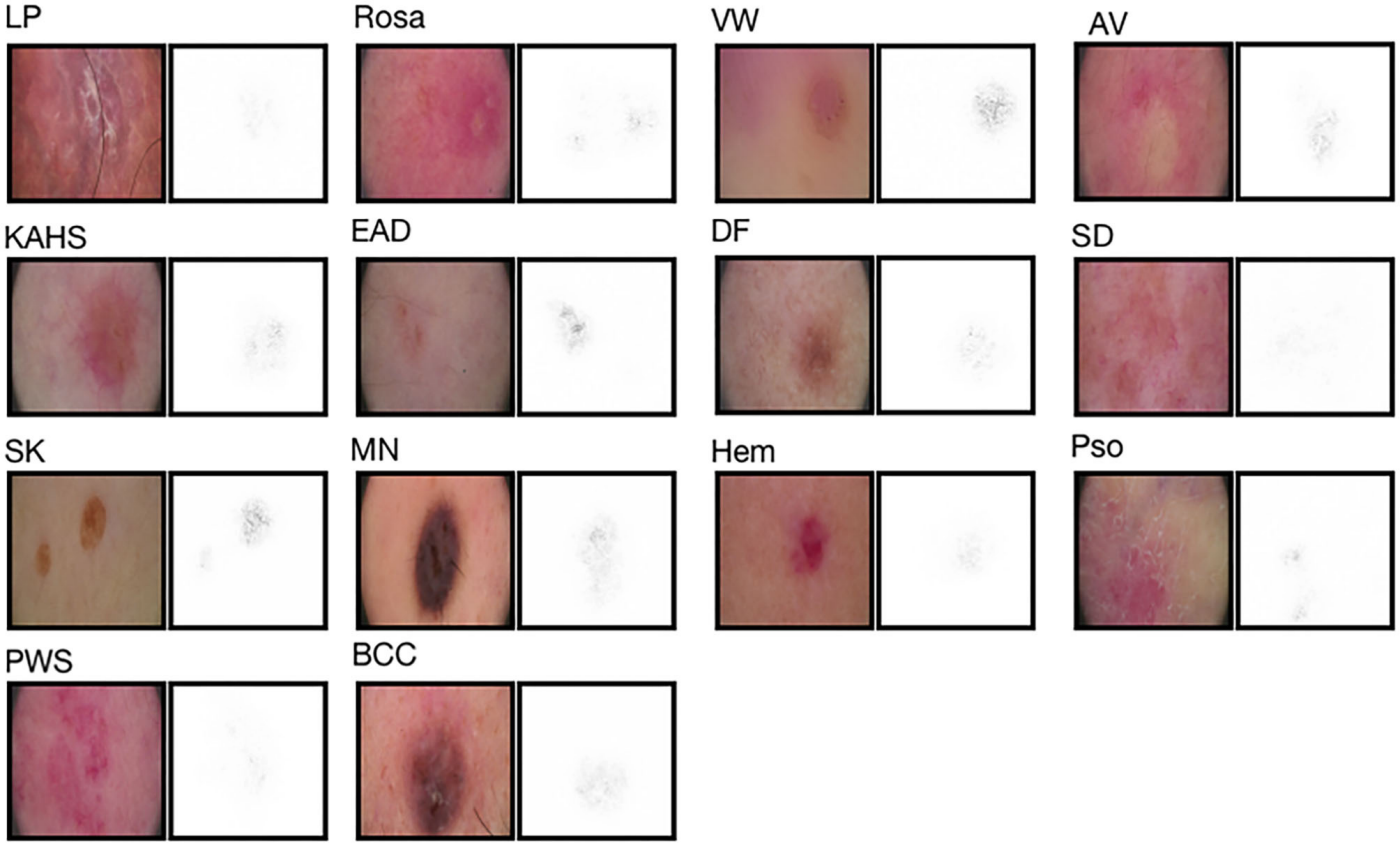
Saliency maps for 14 example images from validation set. Saliency maps for example images from each of the 14 disease classes of the validation set reveal the pixels that most influence a CNN's prediction. Saliency maps show the pixel gradients with respect to the CNN's loss function.

As illustrated in Figure 3, to facilitate triaging referrals and focusing one's clinical examination, we also created a heatmap via gradient-weighted class activation mapping (Grad-CAM) algorithm (20), which can produce visual explanations for CNN based deep learning models. Grad-CAM uses the gradient information flowing into the last convolutional layer to understand the importance of each neuron for a decision of interest thereby highlighting the important regions in the image for prediction. In our case, we used gradients of 14 classification neurons which flowed into the final convolutional layer to produce a coarse localization map highlighting the important regions in the image for predicting the label. It can be noticed that our network has the capability of focusing on the skin area affected by the disease and neglecting the background pixels and healthy skin around the lesions.

### Comparison Between the Proposed CNN Model and Previous Reported Methods

To both quantitatively and qualitatively demonstrate the effectiveness of the proposed deep learning framework, we compared the performance of our algorithm with previous reported methods, namely Inception-v3 (19), ResNet-101 (21), and the original EfficientNet-b4 (16) using our test set.

### Comparison Between our CNN Model and Dermatologists

We also compared the proposed framework with 280 board-certified dermatologists who previously participated in at least 72-h systemic dermoscopic training on diagnosing clinical cases, using an independent test set that consisted of 200 cases with a clinical image and a dermoscopic image. These cases were composed by eight categories of diseases, namely MN, SK, BCC, EAD, SD, Pso, VW and Rosa, with 25 cases each (also demonstrated in Table 1). For this reader study, every dermatologist was asked for the most likely diagnosis of each case from eight choices of included diseases. This questionnaire reflects the actual in-clinic task that dermatologists will decide whether or not to request further examinations or biopsies. For a fair comparison, the proposed deep learning framework also output the top-1 diagnosis with probabilities/confidence scores of the same 8 categories. However, clinical images were not provided to our CNN model. The outcome was considered “Correct” when the diagnosis made by the proposed deep learning framework or a dermatologist was the real diagnosis for the corresponding case.

### Statistical Analysis

In the reader study, Kappa coefficients were used to assess the consistency between dermatologists (or CNN) and the reference standard on the classification of each disease. Kappa coefficient >0.75 indicates good consistency, 0.40–0.75 indicates moderate consistency, and <0.40 indicates poor consistency. Adjusted Z-tests were used to assess differences in Kappa coefficients between dermatologists and CNN. We also calculated the Kappa coefficients of the dermatologists (as standard) and included CNNs. Results were considered statistically significant at the *P* < 0.05 level. All analyses were carried out using Scikit-learn 0.22.2 and Numpy 1.16.4.

## Results

### Performance Evaluation

To evaluate the performance of the proposed framework, we applied our method on a dataset of 13,603 dermatologist-labeled images with 14 categories of skin diseases. Sensitivity and specificity defined below are applied as the performance evaluation metrics:

$$\begin{array}{l} \text{Sensitivity}=\frac{\text{true positive}}{\text{positive}}\\ \text{Specificity}=\frac{\text{true negative}}{\text{negative}} \end{array}$$

**Figure 4:**
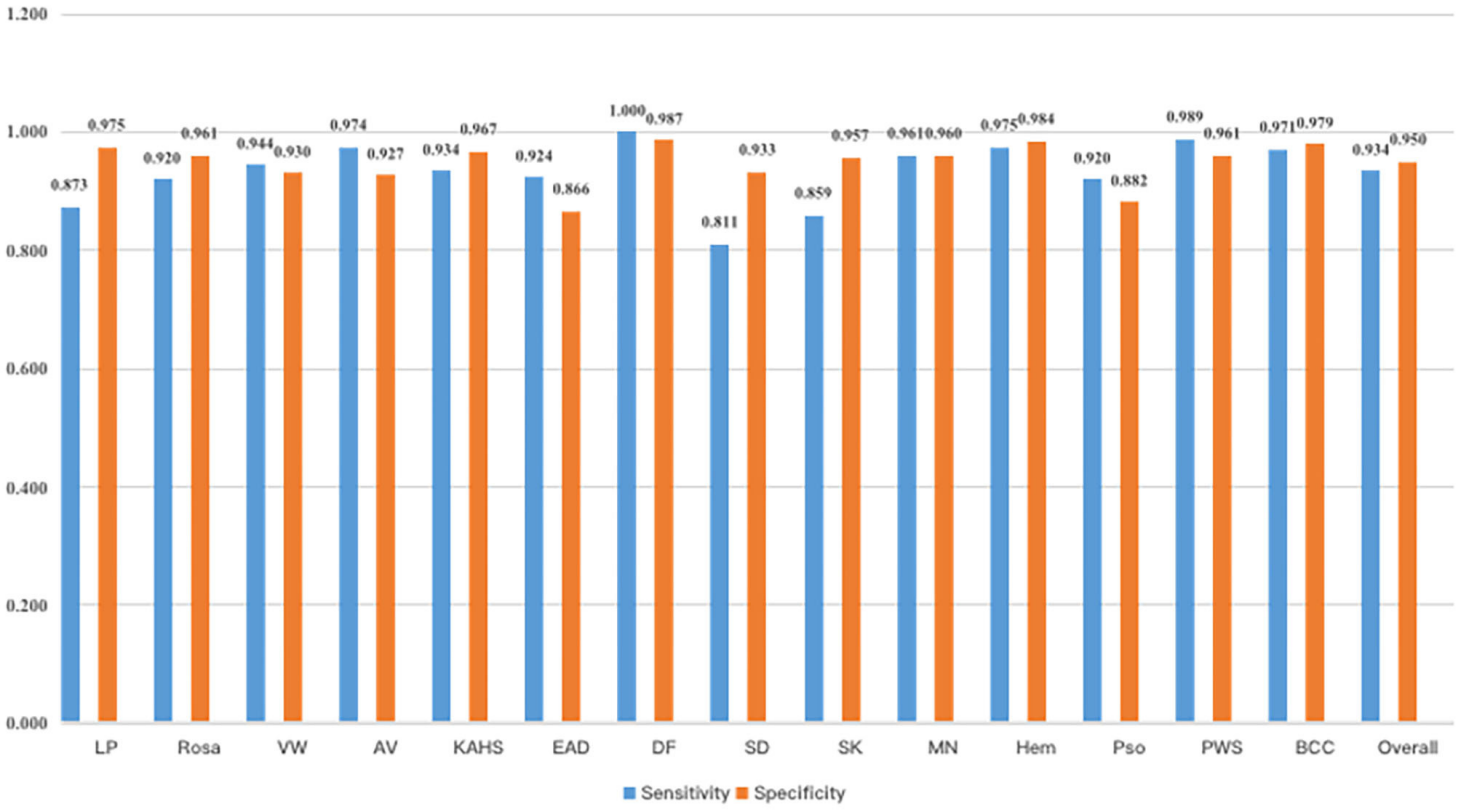
Sensitivity and specificity of our model. As a result, our model had an overall sensitivity of 93.38 ± 0.08% and specificity 94.85 ± 0.05%.

**Figure 5:**
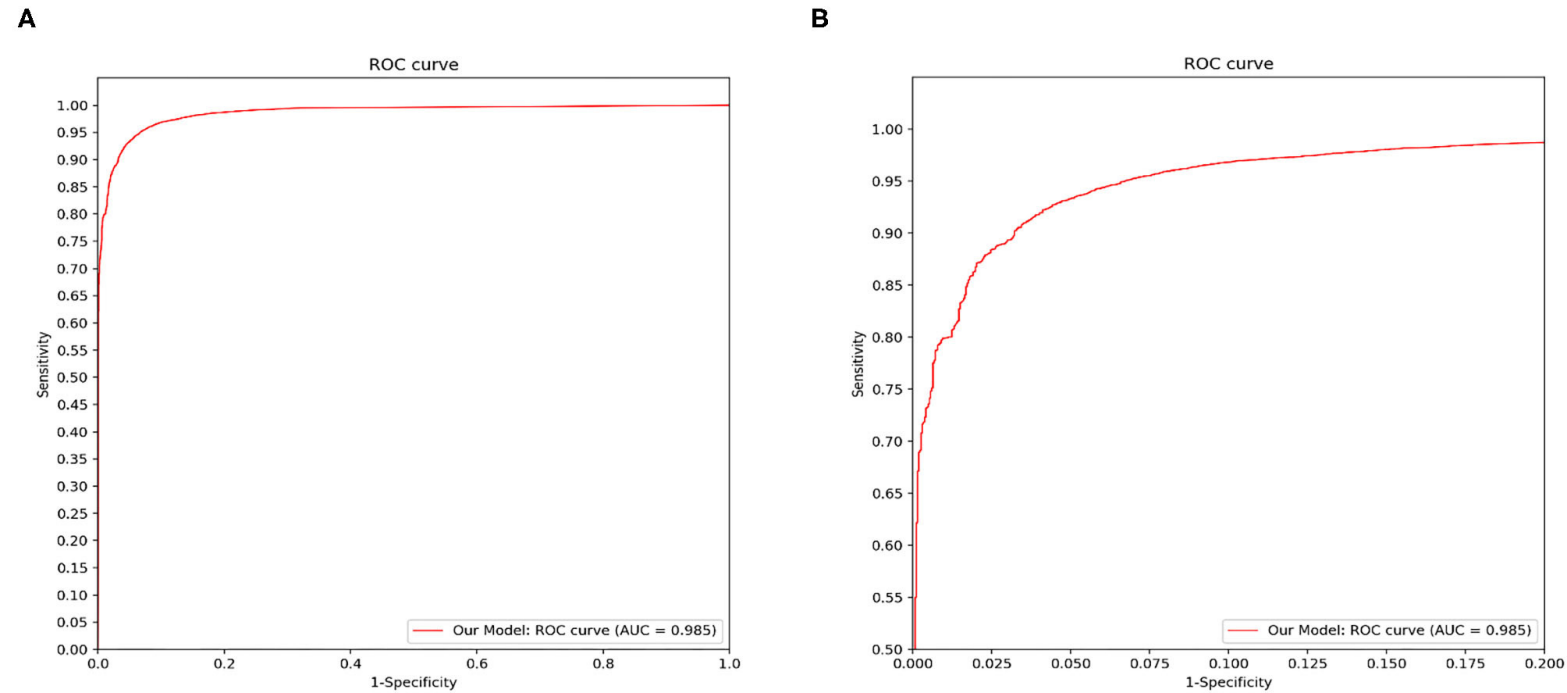
Disease classification performance of the proposed model. We fix a threshold probability t such that the prediction y for any image is y = P ≥ t, and the curve is drawn by sweeping t in the interval 0–1. The AUC is the CNN's measure of performance, with a maximum value of 1. Our model achieves 0.985 AUC in 14-way classification. **(A)** The full view of the ROC curve of the proposed model. **(B)** The local enlarged image of the ROC curve between abscissa 0~0.2.

**Figure 6:**
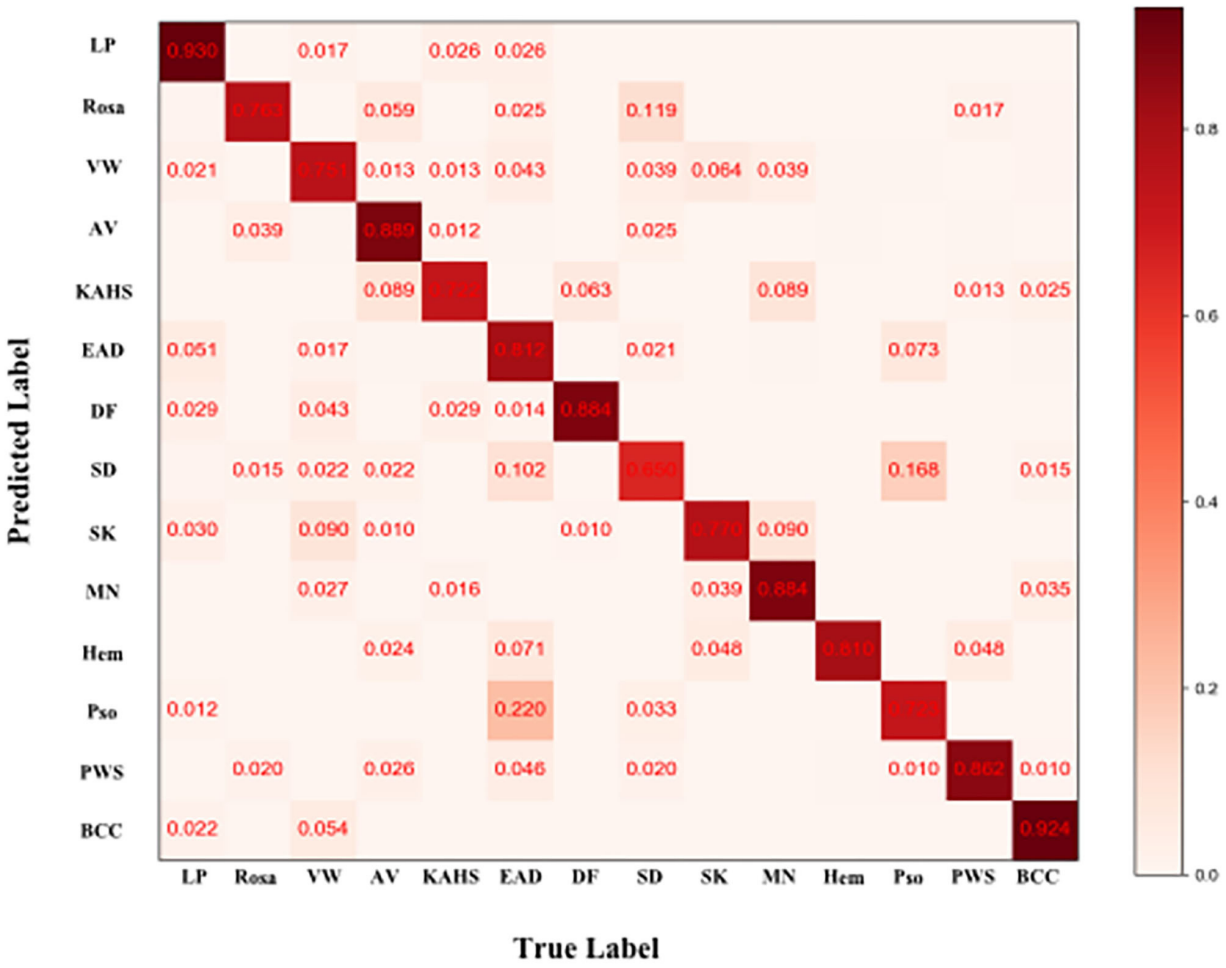
Confusion matrix of the classification result. Element (i, j) of each confusion matrix represents the empirical probability of predicting class i given that the ground truth was class j, with i and j referencing classes from Table 1. Light red means low percentage and deep red represents high percentage.

Figure 4 demonstrated that the proposed framework achieved a rather high overall accuracy of 0.948 ± 0.001 (mean ± SD), with a sensitivity of 0.934 ± 0.001 (mean ± SD), a specificity of 0.950 ± 0.001 (mean ± SD). The specific diagnostic values according to disease categories were summarized in Table 2. Receiver operating characteristic (ROC) curve in Figure 5 also showed that the proposed model achieved 0.985 of area under curve (AUC) value in 14-way classification. Figure 6 illustrated the corresponding confusion matrices for the 14 predefined diseases. The diagonal of the matrix showed the overlap of true positives and ground truth for each label, while the other cells of the matrix were misclassification of images with their true labels.

**Table 2:** The classification accuracy, sensitivity and specificity of the proposed CNN model according to disease category.

**Disease category**	**Accuracy**	**Sensitivity**	**Specificity**
Overall	0.948	0.934	0.950
Lichen planus	0.969	0.873	0.975
Rosacea	0.959	0.920	0.961
Viral warts	0.932	0.944	0.930
Acne vulgaris	0.935	0.974	0.927
Keloid and hypertrophic scar	0.969	0.934	0.970
Eczema/dermatitis	0.877	0.924	0.866
Dermatofibroma	0.987	1.000	0.987
Seborrheic dermatitis	0.926	0.811	0.933
Seborrheic keratosis	0.953	0.858	0.957
Melanocytic nevus	0.960	0.961	0.960
Hemangioma	0.984	0.975	0.984
Psoriasis	0.886	0.920	0.882
Port wine stain	0.963	0.989	0.961
Basal cell carcinoma	0.979	0.971	0.979

### Comparative Outcomes Between the Proposed CNN Model and Previous Reported Methods

**Figure 7:**
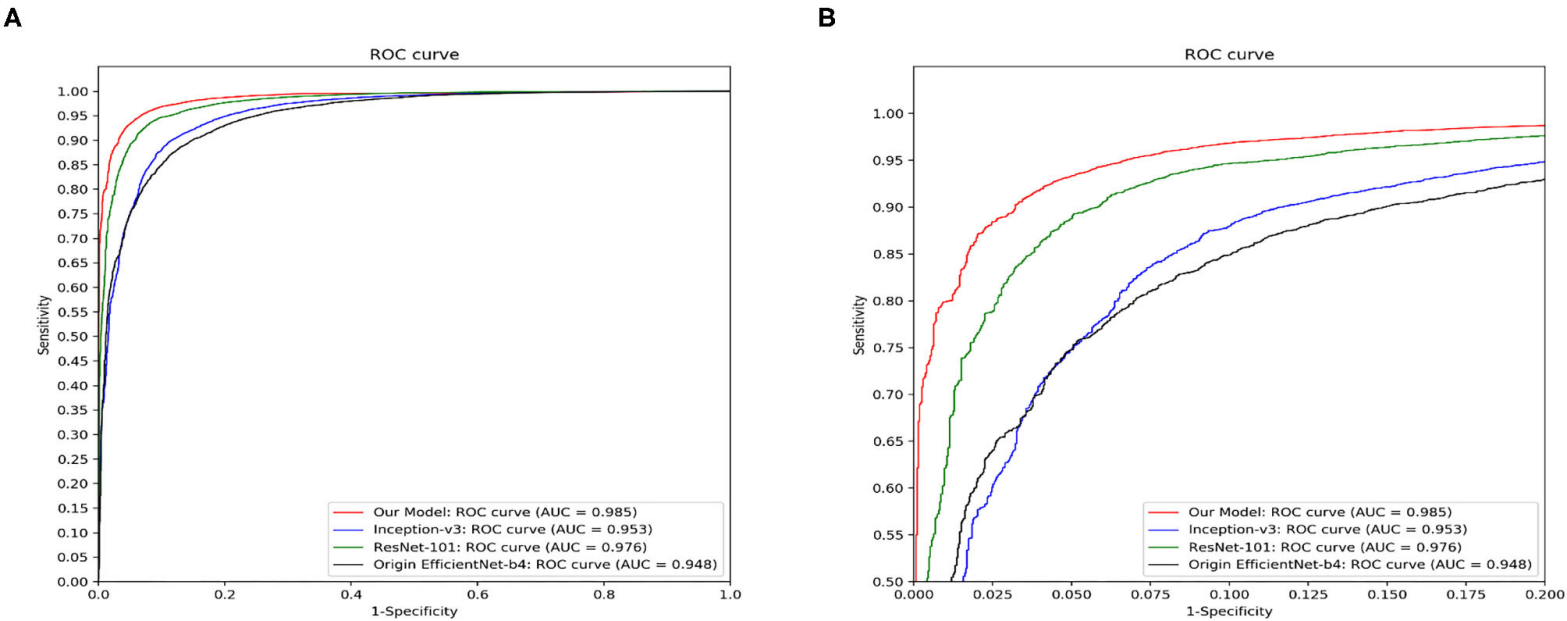
The ROC curves and the AUC value of our model, Inception-v3, ResNet-101 and the original EfficientNet-b4. Our model outperforms other reported methods for this skin disease diagnosis problem. **(A)** The full view of the ROC curve of the proposed model. **(B)** The local enlarged image of the ROC curve between abscissa 0~0.2.

Figure 7 demonstrated the comparative results among our model, Inception-v3, ResNet-101 and the original EfficientNet-b4 with respect to the ROC curves and the AUC value. The AUC value can reflect the overall accuracy of each model. As shown in Figure 7, our model has the best diagnosis performance over all other models. To be specific, our model outperformed existing models with the highest AUC of 0.985 whereas the Inception-v3 had an AUC of 0.953; the ResNet-101 had an AUC of 0.976; and the original EfficientNet-b4 had an AUC of 0.948, respectively, and the ROC curve of our CNN model was always situated higher than the ROC curves of other 3 models. Table 3 revealed the detailed overall sensitivity, specificity and accuracy of our and other 3 CNN models.

**Table 3:** The detailed overall sensitivity, specificity and accuracy of our and other 3 CNN models.

** 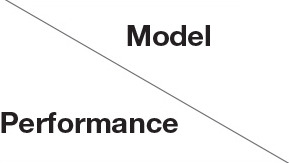 **	**Our model**	**Original** ** EfficientNet**	**ResNet-101**	**Inception-v3**
Sensitivity	0.934	0.882	0.919	0.890
Specificity	0.950	0.875	0.935	0.895
Accuracy	0.948	0.875	0.934	0.895

### Comparative Outcomes Between our CNN Model and Dermatologists

The general information of patients included in an independent dataset is also shown Table 1. The performance of our CNN model and that of the dermatologists using the reader study test set is revealed in Table 4. The proposed CNN model achieved 92.75% average accuracy for all 200 cases, with a sensitivity of 83.50% and a specificity of 94.07%. While the average accuracy of board-certificated dermatologists is 92.13%, with an average sensitivity of 68.51% and an average specificity of 95.50%. Furthermore, according to the Kappa coefficients and adjusted Z test (summarized in Table 5), except for EAD and SD, the CNN model reached better consistency (moderate to good) with the reference standard than the average level of 280 dermatologists, and the difference is statistically significant. It needs to be mentioned that the CNNs is extremely fast, yielding its ranked diagnosis selections within 0.04 s. We also analyzed the diagnostic consistency of dermatologists (as standard) and 4 included CNN models on the 8-class task. Results showed that when taking dermatologists' performance as standard, statistical significance of differences between our model and dermatologists lay merely in psoriasis, and the Kappa coefficient of our model was higher than dermatologists (0.795 vs. 0.675). Furthermore, dermatologists' performance was significantly better than Origin EfficientNet in diagnosing Rosa (0.683 vs. 0.304) and worse in VW (0.533 vs. 0.759). Differences of other classifications between dermatologists and CNNs did not show statistical significance.

**Table 4:** Comparison between doctors and our CNN model in an 8-class task, the better outcomes of our CNN model are colored orange.

**Dermatologists**	**Rosa (%)**	**VW (%)**	**EAD (%)**	**SD(%)**	**SK (%)**	**MN(%)**	**Pso (%)**	**BCC(%)**
Sensitivity	79.70	62.04	74.96	46.53	59.43	77.23	67.66	80.54
Specificity	96.39	98.48	92.42	93.70	94.28	94.68	97.32	96.73
Accuracy	94.31	93.93	90.24	87.81	89.92	92.50	93.61	94.71
**OUR MODEL**								
Sensitivity	92.00	92.00	84.00	48.00	76.00	96.00	92.00	88.00
Specificity	92.57	95.43	88.57	91.43	96.00	94.86	94.29	99.43
Accuracy	92.50	95.00	88.00	86.00	93.50	95.00	94.00	98.00

**Table 5:** Kappa coefficients (95% confidence interval) of the included four CNN models and dermatologists (as standard) on the eight-class task.

**Disease category**	**Dermatologists**	**Our model**	**Original EFFICIENTNET**	**ResNet101**	**Inception-v3**
Rosacea	0.683 (0.609~0.757)	0.712 (0.640~0.783) *P-*value: 0.080	0.304 (0.194~0.414) *P-*value: 0.010	0.646 (0.567~0.726) *P-*value: 0.358	0.691 (0.616~0.765) *P-*value: 0.349
Viral warts	0.533 (0.445~0.621)	0.793 (0.731~0.854) *P-*value: 0.057	0.759 (0.693~0.825) *P-*value: 0.017	0.684 (0.609~0.759) *P-*value: 0.065	0.761 (0.695~0.827) *P-*value: 0.095
Eczema/dermatitis	0.757 (0.694~0.820)	0.570 (0.484~0.655) *P-*value: 0.104	0.507 (0.416~0.598) *P-*value: 0.310	0.558 (0.471~0.645) *P-*value: 0.313	0.585 (0.500~0.670) *P-*value: 0.347
Seborrheic dermatitis	0.607 (0.526~0.689)	0.381 (0.280~0.483) *P-*value: 0.379	0.337 (0.233~0.440) *P-*value: 0.137	0.352 (0.247~0.457) *P-*value: 0.198	0.380 (0.277~0.484) *P-*value: 0.227
Seborrheic keratosis	0.410 (0.311~0.509)	0.708 (0.635~0.780) *P-*value: 0.249	0.576 (0.489~0.663) *P-*value: 0.239	0.576 (0.489~0.663) *P-*value: 0.237	0.682 (0.606~0.758) *P-*value: 0.350
Melanocytic nevus	0.683 (0.609~0.758)	0.799 (0.738~0.860) *P-*value: 0.224	0.786 (0.723~0.849) *P-*value: 0.263	0.759 (0.693~0.825) *P-*value: 0.234	0.719 (0.648~0.790) *P-*value: 0.237
Psoriasis	0.675 (0.600~0.749)	0.759 (0.693~0.825) *P-*value: 0.043	0.744 (0.676~0.813) *P-*value: 0.357	0.621 (0.539~0.703) *P-*value: 0.387	0.701 (0.627~0.774) *P-*value:0.194
Basal cell carcinoma	0.738 (0.671~0.805)	0.905 (0.863~0.948) *P-*value: 0.220	0.875 (0.826~0.924) *P-*value: 0.204	0.725 (0.654~0.796) *P-*value: 0.235	0.853 (0.800~0.905) *P-*value: 0.229

### Visualization of Internal Features

**Figure 8:**
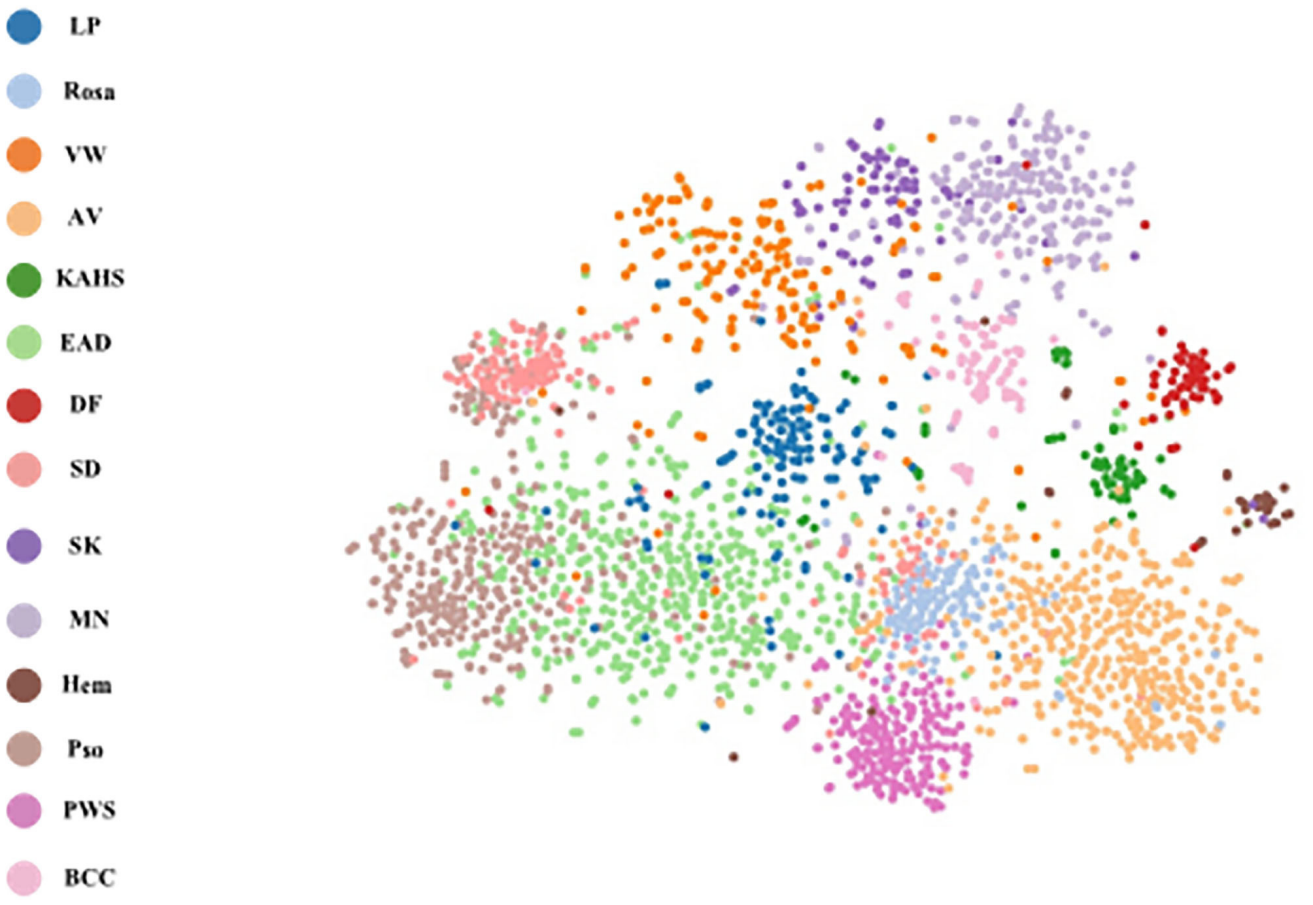
t-SNE visualization of the last hidden layer representations in the CNN for four disease classes. Here we show the CNN's internal representation of eight disease classes by applying t-SNE, a method for visualizing high-dimensional data, to the last hidden layer representation (1792-D vector) in the CNN. Colored point clouds represent the different disease categories, showing how the algorithm clusters the diseases.

To explore the visual characteristics of different clinical classes, we examined the internal features learned by the proposed framework using t-SNE (t-distributed Stochastic Neighbor Embedding) (22). As demonstrated in Figure 8, each point represents a skin image projected from the 1792-dimensional output of the last hidden layer of the proposed network into 2-dimensions. We can notice clusters of points from same clinical classes. This visualization represents that our method is capable of separating various skin diseases objectively for referral.

## Discussion

In China, the board-certificated dermatologists are sparse, with the dermatologist to patient ratio as low as 1:60,000. Furthermore, the majority of well-trained dermatologists are practicing in large cities which worsens the reality that the demand for dermatological consultant is increasingly difficult to satisfy in remote and rural areas of China (23). Moreover, the capacity of giving correct diagnosis and management plans of Chinese dermatologists vary tremendously due to the imbalanced training and learning opportunities of medical education system in China. Although the growing application of multiple non-invasive skin imaging techniques such as dermoscopy, high-frequency ultrasonography and reflectance confocal microscopy has to some extent improved the diagnostic accuracy of Chinese dermatologists, the reality is still not optimistic according to a recent study shown that the imaging diagnostic ability for cutaneous tumors of Chinese dermatologists is relatively poor, and the results of dermatologists in different regions are uneven (24). The insufficiency of well-trained dermatologists and the high incidence of misdiagnosis are calling for a more efficient and accurate way of screening and triaging the Chinese patients suffered from cutaneous diseases.

Ever since Esteva et al. (3) reported that their CNN model, utilizing a GoogleNet Inception v3 CNN architecture trained by a large datasets (>120,000 dermoscopic images), could outperform board-certified dermatologists in classification of skin cancer, the number of researches on AI application in dermatology is constantly increasing and many of them could achieve a dermatologist-level accuracy, giving the hope of improving the primary screening process of patients because it is impossible for all the patients to be referred to the skin cancer professionals before a suspicious diagnosis of malignancy. However, the majority of these researches are based on different imaging datasets that mainly include Caucasian patients with cutaneous tumors, especially malignant melanoma because of its relatively high incidences and risks in Western countries. But in Asia, the prevalence of cutaneous diseases is very distinct from that of Western countries, for example, the average annual age-adjusted incidence rate of melanoma in Asian population was reported as <1/100,000 (25, 26). Therefore, the existing CNN models constructed using a large portion of Caucasian patients' images with a preference of those specific skin diseases can hardly meet the real-world clinical needs in Asian countries like China. In fact, recently, Minagawa et al. (27) confirmed that the diagnostic performance of CNN model in diagnosing skin tumors in Japanese patients would be improved if retrained by a dataset composed of cases with darker skin type.

We developed this CNN model based on pre-trained Google's EfficinetNet-b4 using a unique dataset that consisted of 14 most common skin diseases encountered and examined with dermoscopy in Chinese hospital dermatological clinics, including benign cutaneous neoplasms (MN, SK, DF, and KAHS), cutaneous malignancy (BCC), vascular neoplasm and malformation (Hem and PWS), inflammatory diseases with predilection sites of trunk and extremities (EAD, Pso, and LP) and with predilection sites of facial areas (SD, AV, and Rosa) and infectious diseases (VW). To the best of our knowledge, the construction of this dataset, including 14 categories of diseases, 2,538 cases and 13,603 dermoscopic images, is innovative, with many of the diseases firstly involved in a CNN model training, such as PWS, KAHS, and VW, aiming to propose a CNN model with better adaption of the real, complicated clinical environment in China. We initially chose dermoscopic images to construct the dataset because dermoscopic images usually reveal more valuable information of the lesion morphology than clinical images, and the process of taking a dermoscopic image is easier to standardize, and furthermore the background noises are much more prominent in clinical images which can affect the accuracy of the CNN model. Besides, since each skin diseases included in our research had unique and valuable dermoscopic features for differential diagnosis in clinical practice, we speculated our model would also benefit more from dermoscopic images than clinical images. However, we still tried to perform the 14-classification task using clinical images in the preliminary experiment as well. Results showed that the overall accuracies of clinical-image based CNN was 0.883, while the result our model based on dermoscopic images was 0.948. We thought the modest performance of clinical-image based CNN was relevant to the limited clinical dataset (for each patient, only 1 or 2 clinical images were recorded in our imaging database) and the relatively poor standardization of the clinical images (e.g., complex background interferes, different luminous intensity and camera angles). Therefore, we persisted in presenting our research based on the dermoscopy-based CNN. Yet, we would also attempt to construct CNN models based on the combination of clinical and dermoscopic image data since the former would certainly complement the information such as lesion distributions, skin textures and sites of involvement, potentially contributing to an even more satisfying diagnostic accuracy. The detailed performance results of the clinical-image based CNN in our preliminary experiment on the 14-classification task is also provided within the Supplementary Material.

In a recent article, Liu et al. (28) developed and validated a deep learning system for differential diagnosis of 26 types of skin diseases using clinical-only images from telemedicine, of which the top-1 diagnostic accuracy was non-inferior to dermatologists and higher than primary care physicians and nurse practitioners. Dermoscopy has been increasingly promoted and used in both dermatologists in the metropolises and general practitioners in the rural areas for its convenience and low costs, while the pace of the qualified telemedicine center construction is relatively slower. Therefore, the accessibility of dermoscopy could be better than teledermatology consult in many countries. Furthermore, for inflammatory dermatoses included in our research, each of them has meaningful dermoscopic features for differential diagnosis in clinical practice, well-established and summarized in dermoscopy textbooks, reviews and expert consensuses (29, 30). For example, although clinically similar, Rosa typically appears as multiple polygonal vessels while SD appears as erythema, scattered linear vessels and yellowish scales. According to our experience, CNN might capture those subtle features and make classification more accurate based on dermoscopy images. Moreover, a recent research proposed by Brinker et al. (31) revealed that CNN trained by dermoscopic images could also accurately classify clinical melanoma images, showing that training CNN using images with higher resolution and more details might be able to differentiate images with lower resolution and less information.

Fujisawa et al. (12) previously proposed a CNN model trained by a small dataset of 4,867 clinical images but harvested a satisfactory diagnostic accuracy in classifying 14 skin tumors with an overall accuracy in differentiating benign and malignant conditions of 93.4%. Wang et al. (13) used pre-trained GoogLeNet Inception v3 CNN network trained by 7,192 dermoscopic images also achieved an overall classification accuracy of 81.49% in multiclass model and 77.02% in two-class model. Similarly, considering the classifications are up to 14 categories, the number of images in our dataset is relatively modest, but our CNN model achieved even better results, with an overall sensitivity of 93.38 ± 0.08% and specificity of 94.85 ± 0.05% in this 14-class task, emphasizing that utilizing specific training methods, the CNN model trained by relatively limited imaging data can reach a satisfying performance level as well.

As mentioned above, the proposed CNN model achieved rather high overall classification accuracy, sensitivity and specificity, and was considered capable of aiding the patient screening in real dermatological clinics of China, especially for the remote and rural areas where the medical resources were extremely limited. We also compared our CNN model with previous reported methods including Inception-v3, ResNet-101, and the original EfficientNet-b4. Results showed that our model outperformed all of them in ROC curve and AUC value evaluation using our test set images.

For further verifying the effectiveness of this proposed CNN model, we compared it with 280 board-certificated dermatologists who undergone at least 72-h systemic dermoscopic training, using an independent test dataset different from that for the routine performance evaluation process. To the best of our knowledge, this was the largest group of dermatologists included to compare with AI performance (10, 12, 13, 32–34). Results demonstrate that our CNN model has higher sensitivity in all the tested eight categories of skin diseases compared with dermatologists, and higher accuracy in five of them (namely VW, SK, MN, Pso, and BCC), higher specificity in 3 of them (namely, SK, MN and BCC). And statistical analysis revealed that the proposed CNN model reached better consistency (moderate to good) with the reference standard than the average level of 280 dermatologists in 6 of the 8 categories (namely Rosa, VW, SK, MN, Pso, and BCC), and the difference is statistically significant. However, the dermatologists had better outcomes in EAD and SD. According to the confusion matrix of the classification result (Figure 6), the proposed CNN model misclassified EAD mostly into Pso and SD, and misclassified SD mostly into Rosa. In our training set, the numbers of Pso images (1707) and EAD images (2440) are much larger than SD images (767) and Rosa images (597), which could lead to an imbalance of data and brought a risk of this inconsistency. Furthermore, in real clinical practice, the diagnosis of EAD is quite straightforward, based on the clinical lesion morphology and symptoms like pruritus in China, including the cases of our dataset, so the morphological diagnosis might include various EAD subtypes (e.g., dyshidrotic, disseminated and nummular) and disease durations (acute, subacute, and chronic), which would at last largely affect the feature extraction of EAD for the CNN. Moreover, it is often quite difficult to differentiate these inflammatory dermatoses in real clinical practice and the conditions of coexistence are not rare. Of note, the dermatologists were even provided with extra clinical images because this process would be more like the real clinical environment. This is critical because for inflammatory dermatoses like EAD, Pso, SD, and Rosa, the lesion distribution and sites of involvement that only shown in clinical images are of vital importance for differential diagnosis, which could also contribute for the relatively worse performance of our CNN in classifying EAD and SD. The outcomes reveal that our CNN model is dependable in classifying theses common cutaneous conditions, and might have a fewer chance to miss the correct diagnosis compared with dermatologists.

However, our study has several limitations. First, our dataset completely came from the imaging database of the Department of Dermatology, Peking Union Medical College Hospital, using the same dermoscopy system. Therefore, it is possible that the accuracy of our CNN model might be lower when the dataset involves cases and images from other hospital or devices because the standardization would be affected. Second, as for the real clinical practice, the diagnosis of cutaneous diseases is not only based on the morphology of the skin lesions but also impacted by the general information and complicated medical history of the present patients, but we still do not know whether the accuracy would increase or decrease if such additional information is provided to the CNN model. Third, the disease spectrum in dermatology is rather wide. Although our dataset could to the best extent mimic the overall clinical conditions in our daily practice, there still lack a vast range of cutaneous diseases, such as skin cancers (e.g., squamous cell carcinoma and melanoma) uncommon in China. For further study, a CNN model should be refined by utilizing data from multiple centers or devices to improve the universality, and including more constantly encountered disease types in clinical practice, and possibly adding the information other than dermoscopic images such as sex, age, disease duration and clinical or high-frequency ultrasonic images. Finally, as forwarded by Tschandl et al. (35), CNN-support diagnosis gains are relative to the clinicians' experience, confidence in diagnosis and specific tasks. Human–computer collaboration will make full use of CNN assistance. The future studies ought to emphasize the cooperation instead of competence between clinicians and CNN. However, our study lacked the validation of the influences of CNN assistance on dermatologists as mentioned above.

In conclusion, we proposed a CNN model based on Google's EfficientNet-b4 with pre-trained weights on ImageNet trained by a novel dermoscopic dataset represented the real dermatological clinics environment of a tertiary class hospital in China with 14 categories of common cutaneous diseases. Our CNN model achieved a rather high level of performance, with an overall accuracy of 0.948 ± 0.001 (mean ± SD), a sensitivity of 0.934 ± 0.001 (mean ± SD), and a specificity of 0.950 ± 0.001 (mean ± SD). Furthermore, we compared this framework with previously reported methods and it outperformed all of them. Also, the performance of this CNN model was comparable to 280 board-certified dermatologists in an eight-class diagnostic task, with higher sensitivity in all of the included diseases.

## Data Availability Statement

The datasets for this study are not available because they contain the dermoscopic images of patients with cutaneous diseases according to the regulations of our informed written consents. Requests to access the datasets should be directed to Jie Liu, liujie04672@pumch.cn.

## Ethics Statement

The studies involving human participants were reviewed and approved by the Medical Ethics Committee of Peking Union Medical College Hospital. Written informed consent to participate in this study was provided by the participants' legal guardian/next of kin.

## Author Contributions

C-YZ, Y-KW, H-PC, K-LG, and JL made the concept and design of this study. C-YZ, Y-KW, and JL were responsible for the literature search, patient enrolment, dermoscopic evaluation, and dataset formation. H-PC, L-FY, and K-LG developed, refined, and validated the proposed CNN model using the dataset mentioned above. C-YZ, Y-KW, CS, J-CW, and JL organized the comparative reader study of 280 board-certificated dermatologists in a formal dermoscopy conference in China. Y-GY and F-YX collected and analysed the classification results of dermatologists. H-PC and Y-GY made the statistical analysis. The manuscript was prepared by Y-KW and H-PC, edited and reviewed by L-FY, JL, and F-YX. All authors contributed to the article and approved the submitted version.

## Conflict of Interest

The authors declare that the research was conducted in the absence of any commercial or financial relationships that could be construed as a potential conflict of interest.
