# Supplementary material for: A Deep Learning Based Framework for Diagnosing Multiple Skin Diseases in a Clinical Environment
Source: Front Med (Lausanne). 2021 Apr 16;8:626369. doi: 10.3389/fmed.2021.626369 (PMC8085301; doi:10.3389/fmed.2021.626369)
Supplement: Supplementary file 1 [file Table_1.docx]

| Data    Performance | Dermoscopic Image | Clinical Image |
| --- | --- | --- |
| Sensitivity | 0.934 | 0.880 |
| Specificity | 0.950 | 0.883 |
| Accuracy | 0.948 | 0.883 |

**Supplementary Material**

Supplementary material 1. CNNs performance of different data sources in the 14-classification tasks using the validation set.
